# Engagement in sex work does not increase HIV risk for women who inject drugs in Ukraine

**DOI:** 10.1093/pubmed/fdw070

**Published:** 2016-07-22

**Authors:** Tetyana I. Vasylyeva, Samuel R. Friedman, Lenore Gensburg, Pavlo Smyrnov

**Affiliations:** 1 Department of Zoology, University of Oxford, Oxford OX1 3PS, UK; 2 National Development and Research Institutes, New York, NY 10010, USA; 3 School of Public Health, State University of New York at Albany, Albany, NY 12222, USA; 4 International HIV/AIDS Alliance in Ukraine, Kyiv 03680, Ukraine

**Keywords:** epidemiology, public health, sexual behavior

## Abstract

**Background:**

We studied the association between sex in exchange for money, drugs or goods and HIV for women who inject drugs (WWID) in Ukraine, as previous data on this association from the post-USSR region are contradictory.

**Methods:**

Data come from the Integrated Bio-Behavioral Survey of Ukrainian people who inject drugs collected in 2011 using respondent-driven sampling. Participants were interviewed and tested with rapid HIV tests.

**Results:**

The sample included 2465 WWID (24% HIV positive); 214 (8.7%) of which reported having had exchange sex during the last 90 days. Crude analysis showed no association between exchange sex and HIV (OR = 0.644; 95% CI 0.385–1.077). No confounders were found to alter this result in a multivariable analysis. Further modeling showed that exchange sex modifies association between HIV and alcohol use: no association between HIV and daily alcohol use was found for those women who exchanged sex (OR = 1.699, 95% CI 0.737–3.956); while not engaging in sex work and daily using alcohol reduced odds to be HIV infected (OR = 0.586, 95% CI 0.389–0.885).

**Conclusions:**

Exchange sex may have less impact on the HIV status of WWID who are exposed to injecting risks. The finding that daily alcohol use appears protective against HIV among WWID who do not exchange sex requires more research.

## Background

The HIV epidemic in Ukraine is one of the most severe in Europe. In 2011, there were 21 177 people newly diagnosed with HIV (46.2 per 100 000 population)—the highest number since the start of HIV monitoring in Ukraine in 1987.^1^ Still, about a half of those infected are unaware of their HIV status—indicating a big gap in HIV testing.^2^ Traditionally, people who inject drugs (PWID) have been the main driving force of the HIV epidemic in the country. Before early 2000s up to 93% of Ukrainian PWID practiced some of the unsafe injecting behaviors,^3^ which allowed easy spread of HIV. Parenteral route remained the main mode of HIV transmission until 2008 when Ukraine observed more cases of sexually transmitted HIV. In 2011, the percentage of newly diagnosed people who reportedly contracted HIV through sexual contact increased to 49%, while parenteral transmission was 31%.^1,4^ Since then the HIV epidemic in Ukraine is no longer concentrated in marginalized groups of PWID or sex workers, the estimates show 1.6% of adult population (14–65 years old) to be infected with HIV.^5^

Supposedly, this generalization might be driven by so-called ‘bridge’ groups that connect social groups with high HIV prevalence (such as PWID) and groups with low HIV prevalence (general population). One such group is women who inject drugs (WWID) who also engage in exchange sex (sex in return for money, drugs or goods). WWID who exchange sex have a potential for HIV transmission from their injecting to their non-injecting sexual partners, who in their turn can transmit it to their other non-injecting sexual partners. Studies from Russia show that some 15–50% of WWID exchange sex to receive money for drugs and that ~25–80% of female sex workers (FSW) in Russia inject drugs.^6^ Data from Togliatti City, Russia, show that exchange sex is associated with increased odds for sexually transmitted diseases (STDs) among WWID, which might be a marker of on-going sexual transmissions of HIV.^7,8^ Data from Ukrainian Integrated Bio-Behavioral Surveys (IBBS) indicate that in 2011 10% of FSWs reported injecting drugs and 20% of WWID reported having exchange partners.^1,9^ However, the actual numbers might be higher.

WWID who exchange sex are at elevated risk for HIV due both to risky sexual and injection practices. Some of the various risky sexual behaviors are independently associated with HIV, including increased number of partners,^10,11^ increased prevalence of STDs^6,11–13^ and being a victim of violence.^14–16^ Additionally, a study of young PWID in San Francisco showed that WWID are more likely to borrow needles and to be injected by other PWID than male PWID.^17^ The authors hypothesized that this behavior was associated with having an injecting sexual partner, indicating a dependence of WWID on their male sexual injecting partners.

During the generalization of an epidemic, bridge groups play the leading role in the spread of HIV. Learning what are their risks and how those risks can be reduced through targeted preventive interventions is of great public health importance. In this paper, we investigated if exchange sex imposes greater risk for being HIV positive for WWID controlling for other factors.

## Methods

### Sampling

Data come from the IBBS of PWID (*n* = 9069) conducted in 26 cities of Ukraine in 2011. Respondent-driven sampling (RDS) was used to sample participants.^18^ RDS is a non-probability sampling technique, an extension of the chain-referral sampling, known for its ability to sample hard-to-reach populations. Sampling starts with several initial respondents called ‘seeds’ who are interviewed and given three coupons to invite their PWID peers to be interviewed. When recruits come, they are interviewed and given three coupons. This continues until recruitment chains are long enough to converge to a sampling equilibrium.^19^ Data weighting according to the size of a social network of every participant is often used to make results generalizable.  Here we did not use RDS weighting since we have only used a sub-sample of the full survey sample and it is not yet known how to weight for a sub-sample from within the recruitment chains of an RDS process.^19^ Eligibility criteria for participants in the survey included: (i) age older than 14; (ii) drug injecting over the last 30 days and (iii) residence or long-term stay in the city where the survey takes place.

In IBBS-2011 participants were interviewed about their behavior (injecting and sexual practices), HIV and STD history, attitudes and knowledge about HIV, and participation in harm reduction projects. After the interview every participant underwent a rapid HIV test.

Bioethical approval of the IBBS-2011 survey was obtained at the Institute of Epidemiology and Infectious Diseases of the Academy of Medical Sciences of Ukraine.

### Statistical analyses

The outcome of primary interest was HIV test result (positive/negative) as measured by the rapid test at the end of the survey. The exposure to exchange sex was defined by a question ‘In the last 3 months, did you have any partners who gave you drugs, money or goods in exchange for sex?’ The unexposed category (those who reported no exchange partners in the last 90 days) included those who reported no sex partners of any type in the last 90 days.

Additional covariates that could affect the association of interest according to existing literature were: socio-demographic factors (age, education, employment), risky injecting practices (unsterile syringes/needles, frequent injections, duration of injection), risky sexual practices (not using a condom at last sex, number of sexual partners over the past 3 months) and other substance use (alcohol use and/or use of non-injecting drugs in the past 30 days). Risky injecting/sexual practices and substance use were defined by the answers to a set of questions.

Injecting practices:

– Have you used a new sterile syringe the last time you injected drugs?
– Have you used a syringe that someone else has used previously in the last 30 days?
– Have you used a syringe filled from someone's used syringe in the last 30 days?
– Have you used a prefilled syringe, when you didn't see how the syringe was filled in the last 30 days?
– How often did you share a cooker in the last 30 days?
– How often did you fill in a syringe from a shared container in the last 30 days?

Sexual practices:

– Have you used a condom the last time you had sex?
– How many people did you have a vaginal or anal intercourse within the last 90 days?

Substance use:

– Have you used alcohol in the last 30 days?
– If yes, how often did you use alcohol in the last 30 days?
– Have you used any non-injecting drugs in the last 30 days?

For all of the variables, we have treated the ‘Don't remember’ and ‘Cannot tell’ categories as missing data.

In this analysis, a sub-sample of IBBS participants was used. Only females who were either HIV negative or who first learned about their HIV status in this survey were included. We excluded those who were aware of their HIV-positive status before participation in IBBS to avoid ‘reverse causation effect’, which is likely to occur in the situation of a well-established HIV epidemic like Ukrainian. Particularly, we were afraid that some women could acquire HIV through exchanging sex and cease exchanging sex once they became aware of their HIV-positive status. Including them could lead to a misleading finding that exchange sex is protective against HIV.

Univariate statistics were used to describe our sub-sample. Then bivariable analysis using the *χ*^2^-test was performed to check for associations between:

– exchange sex and HIV,
– factors that might be associated with HIV and thus potentially confound the relationship between exchange sex and HIV,
– factors that might be associated with exchange sex and thus potentially confound the relationship between exchange sex and HIV.

According to a classic definition of confounding given by Rothman *et al*.,^20^ a variable is considered to be a confounder if it is associated with an exposure, a disease and is not on the causal pathway between the two. Only variables that met these criteria (and thus were associated in the bivariable analysis both with exchange sex and with HIV at the *P* = 0.1 level of significance) were included in the multivariable analysis. The initial multivariable regression model included the exposure variable, all the variables that met the criteria in the bivariable analysis plus an interaction term between exchange sex and alcohol use in the last 30 days as studies show that alcohol use is associated with unsafe sex, particularly in HIV-infected women.^21^ In the further process of model building, variables were excluded if they did not change the association between HIV and exchange sex by 10% or more.

Statistical analysis was performed using SPSS version 16 for Windows.

## Results

In IBBS-2011, 9069 PWID were surveyed; 27.5% of them were women (2492). The final sample excluded women who knew about their HIV positive status before (27, 1% of all women). Socio-demographic characteristics of the sample are presented in Table 1. The final sample included 2465 WWID who were either HIV negative (1880, 76%) or who first learned about their HIV positive status in IBBS-2011 (585, 24%). Among those 2465 WWID that were included in the analysis, 214 (8.7%) reported having had exchange sex during the last 90 days. Those 2251 women who did not exchange sex included 225 women who reported no sexual partners in the past 90 days (9% of those 2465 WWID included in the analysis). The median age of the sample was 31 years old, ranging from 14 to 64 years old.

**Table 1:** Socio-demographic characteristics of participants included in the analysis.

*Variable*	*IDU-2011*	
	N	*%*
Age		
14–24	544	22.0
25+	1922	78.0
Marital status		
Live with husband/wife/other sexual partner	1509	61.3
Have a partner, but live alone	71	2.9
Single	881	35.8
Education		
<Complete school	426	17.3
Complete school (11 grades)	1366	55.5
Basic secondary education (technical college)	463	18.8
Complete secondary education (bachelor, master, PhD)	206	8.4
Employment		
Employed	442	18.0
Occasionally employed	675	27.4
Unemployed	698	28.4
Other (pupil, student, household keeper, disabled, maternal leave, retired)	644	26.2
Type of the main drug used		
Opiates	1801	73.1
Stimulants	626	25.4
Other	37	1.5
Reported exchange sex in the past 90 days		
Yes	214	8.7
No	2018	82.1
Did not ask	225	9.2
Client of harm reduction project		
Yes	804	32.6
No	1659	67.4
HIV status		
Positive	585	23.7
Negative	1880	76.3

Variables that met the definition of confounders in the bivariate analysis are listed in Table 2. The number of sexual partners in the last 90 days deemed to be in the pathway between HIV status and exchange sex because those who have exchanged sex in the last 90 days tend to have more partners and more partners is associated with higher risk for HIV. Additionally, since age and duration of injection were highly correlated, only duration of injection was included in the initial logistic regression model since it is a good measure of potential HIV exposure through injection.

**Table 2:** Bivariate analysis of factors that are associated with HIV and exchange sex.

*Variable*	*HIV positive,* N *(%)*	*HIV negative,* N *(%)*	χ^2^-*test,* P-*value*	*Exchanged sex,* N *(%)*	*Did not exchange sex,* N *(%)*	χ^2^-*test,* P-*value*
Age						
14–24	33 (11)	493 (27)	<0.001***	80 (37)	462 (21)	<0.001***
25+	260 (89)	1362 (73)		134 (63)	1782 (79)	
Employment						
Employed	74 (25)	323 (17)	0.002	29 (14)	413 (18)	<0.001***
Occasionally employed	59 (20)	529 (29)		97 (45)	573 (26)	
Unemployed	82 (28)	525 (28)		52 (24)	645 (29)	
Other (pupil, student, household keeper, disabled, maternal leave, retired)	78 (27)	473 (26)		36 (17)	607 (27)	
Alcohol use last 30 days						
Every day	15 (5)	175 (10)	0.001**	26 (14)	139 (8)	<0.001***
1–2 times/week	95 (33)	739 (40)		82 (46)	700 (40)	
1–2 times/month	88 (30)	495 (27)		48 (27)	500 (28)	
No	91 (32)	422 (23)		23 (13)	427 (24)	
Non-injection drug use last 30 days						
Yes	90 (31)	743 (40)	0.002**	91 (49)	674 (38)	0.002**
No	204 (69)	1108 (60)		94 (51)	1109 (62)	
Duration of injecting						
<3 years	25 (9)	355 (19)	<0.001***	23 (13)	326 (18)	0.048*
≥3 years	268 (91)	1488 (81)		161 (87)	1449 (82)	
How often did you share cooker (last 30 days)?						
Always	60 (21)	442 (24)	0.020*	19 (10)	455 (26)	<0.001***
Sometimes	103 (35)	736 (41)		93 (51)	683 (39)	
Never	127 (44)	643 (35)		72 (39)	615 (35)	
How often did you fill in syringe from shared container (last 30 days)?						
Always	60 (21)	427 (23)	0.041*	23 (12)	434 (25)	0.001**
Sometimes	97 (33)	697 (38)		86 (47)	651 (37)	
Never	135 (46)	702 (39)		75 (41)	674 (38)	
Age of sexual debut						
≤16	133 (45)	730 (40)	0.106*	139 (76)	1047 (59)	<0.001***
>16	159 (55)	1099 (60)		44 (24)	734 (41)	
Frequency of sexual contacts last 90 days						
Once or week or less	95 (33)	639 (35)	0.013*	33 (18)	698 (39)	<0.001***
2–6 times per week	118 (41)	867 (47)		97 (53)	887 (50)	
Once a day or more	45 (15)	194 (10)		52 (29)	188 (11)	
No contacts	31 (11)	142 (8)				
Number of sexual partners last 90 days						
1–2	228 (79)	1416 (77)	0.067*	9 (5)	1632 (92)	<0.001***
3–10	20 (7)	187 (10)		76 (43)	131 (7)	
11+	10 (3)	96 (5)		93 (52)	13 (1)	
No contacts	31 (11)	142 (8)				
Client of harm reduction project						
Yes	114 (39)	516 (28)	<0.001***	72 (39)	526 (30)	0.008**
No	180 (61)	1339 (72)		113 (61)	1260 (70)	

*Indicate *P*-value <0.1; **P < 0.01; ***P < 0.001. In bald—variables that were significantly associated both with exchange sex and HIV. Data might not add up to 2465 as some of the variables had missing data.

In the crude analysis, exchange sex was inversely associated, though not significantly, with HIV status, OR = 0.644 (95% CI 0.385–1.077). In the multivariable logistic regression model, the interaction between exchange sex and alcohol use was statistically significant (*P* < 0.001). Thus, further analyses were stratified by reported alcohol use behavior (‘daily alcohol use’ versus ‘occasional or no alcohol use’), but no association between exchange sex and HIV was observed for either category of the alcohol use. In the further process of model building, no other variables were observed to change the association between exchange sex and HIV significantly (for more than 10%) for any of the categories of the alcohol use.

Given that the alcohol use and exchange sex interaction term was significant in the analysis, but no difference in the association between HIV and exchange sex was found between the strata of alcohol use variable, it was decided to explore if exchange sex alters the association between the alcohol use and HIV. In the crude analysis, daily alcohol use seemed to significantly reduce the odds of WWID to be HIV positive (OR = 0.518, 95% CI 0.301–0.891, Table 3). We further performed a bivariable analysis of association between alcohol use and HIV status within the categories of exchange sex to find the explanation of the interaction between exchange sex and alcohol use in their association with HIV. The results of these analyses are also presented in Table 3. For those who did not exchange sex, daily alcohol use kept its significant protective effect (OR = 0.586, 95% CI 0.389–0.885). However, for those WWID who used alcohol daily and exchanged sex, the odds for HIV were non-significantly higher compared to those who used alcohol occasionally or did not use it at all in the last 30 days (OR = 1.699, 95% CI 0.737–3.956).

**Table 3:** Results of bivariate analysis of association between alcohol use and HIV stratified by exchange sex.

*Variable*	N	*OR*	*95% CI*
Daily alcohol use	214	0.518	0.301–0.891
Occasional alcohol use or no alcohol use	2016	1	
*WWID who exchange sex (total 208 with the information on alcohol use)*			
Daily alcohol use	36	1.699	0.737–3.956
Occasional alcohol use or no alcohol use	172	1	
*WWID who do not exchange sex (total 2022 with the information on alcohol use)*			
Daily alcohol use	178	0.586	0.389–0.885
Occasional alcohol use or no alcohol use	1844	1	

## Discussion

### Main finding of this study

Our analysis showed that exchanging sex for money, drugs or goods is not associated with increased HIV risk for WWID in Ukraine. Furthermore, exchange sex seems to interact with alcohol use in WWID. Some studies have previously reported that alcohol use is associated with exchange sex and HIV risk among women,^22^ but no such research was done in WWID. In our analysis, daily alcohol use was inversely associated with the frequency of injection, which might explain its protective effect for those WWID who do not exchange sex. On the contrary, for WWID who exchange sex reduced injecting risks associated with lower frequency of injection might have been complemented with increased sexual risks associated with alcohol use. We suggest that the interaction between alcohol use, injecting and sexual risks among WWID needs further research. Harm reduction programs for WWID need to include safe alcohol (as well as injecting drugs) use messages into educational materials.

### What is already known on this topic

In Russia, a country with a similar HIV epidemic profile, studies are inconsistent with respect to the relationship between exchange sex and HIV. A prospective cohort study from St. Petersburg demonstrated a positive association between exchange sex and HIV status in bivariable, but not multivariable analyses (when controlled for psychostimulants use).^10^ However, this study included males as well as females in the analysis, which might be misleading since at least some of the males might report MSM (men who have sex with men) contacts when asked about exchange sex and the associations between exchange sex and HIV might be different for MSM and heterosexual contacts. One survey of PWID (both male and female) from Togliatti City showed that those exchanging sex are 2.5 times as likely to be HIV positive as those PWID not exchanging, controlling for other factors: year of study, sex, district of residence, education, main source of income, duration of drug use and frequency of injection.^23^ Another study of PWID from three Russian cities reported no association between exchange sex and HIV for female injectors, but there were only seven HIV positive cases and they probably had not enough power to detect the association even if it exists.^24^

### What this study adds

No support was found for the hypothesis that exchange sex increased the odds of HIV seropositivity among WWID in Ukraine. This finding might be counter-intuitive, as exchange sex has been shown before to increase HIV odds.^25^ However, we think that exchanging sex might be less HIV exposing than injecting drugs for WWID in Ukrainian settings, if the exchange sex customers are not PWID.

Surprisingly, duration of injecting drug use did not meet the definition of confounder, even though other studies reported that it changes the association between exchange sex and HIV.^12,23^ Some studies found that the association between exchange sex and HIV among WWID was present only in recently initiated injectors,^23,26^ suggesting that the effect of sex work might be present only when exposure to drugs was short. This might be true for this analysis as well, but this was impossible to test since reducing the sample only to women who use drugs for <3 years made the sample of HIV positive WWID who exchange sex too small.

The fact that exchange sex and alcohol use interplay to change the association with HIV might help to explain the contradictory results of other studies. Alcohol use itself was a risk factor for HIV for Ukrainian WWID in another study.^27^ Both alcohol use and exchange sex are associated with higher numbers of sexual partners for WWID, which is a well-known risk factor for HIV.^28^ Thus, the protective effect of alcohol for those WWID who do not exchange sex was surprising, but might be attributed to the fact that in our analysis alcohol is inversely associated with frequency and duration of drug injection. Consequently, the daily alcohol use may be an indicator of shorter exposure to drug injecting and in the absence of exchange sex it also does not significantly increase sexual risk behaviors.

### Limitations of this study

This is a secondary data analysis and the original survey was not designed to answer the question of the relationship between exchange sex and HIV. Therefore, some of the important questions were not asked. For example, participants were first asked if they had casual partners and then if they used condoms with casual partners (yes/no); the same for main and exchange partners. Consequently, participants who had all types of partners had different answers (yes/no) for different types of partners. Thus, the only variable that could provide information for all the participants was condom use at last sex, but it is known to be a less accurate measure and could leave residual confounding.^29^

The self-reported nature of the data gives us a reason to interpret the answers carefully. Particularly, we are aware that many of the participants are clients of harm reduction projects and were many times ‘taught’ the right answers, especially to the questions about risky behaviors. However, research has shown that self-reported data on drug use and HIV risk behaviors from PWID tend to be reliable and valid.^30^

Despite these limitations, this was the first study that was specifically focused on the examination of the association between exchange sex and HIV among WWID in the region of Eastern Europe, where exchange sex and drug use are prevalent and correlated. Furthermore, since it had a larger sample (2466 WWID) in the analysis than other research in the region, it was possible to study effect modifiers.

## Conclusion

Although exchanging sex was not associated with increased HIV risk in Ukraine, there are subgroups of WWID who may be at increased risk. Research on the interaction of alcohol and exchange sex among WWID is clearly needed so we can understand the apparent protective effect of alcohol use for some WWID and risk of daily alcohol consumption for others.

## Funding

We gratefully acknowledge support from National Institutes of Health (grant number D43TW000233), the Fogarty International Center and the NIDA (grants P30 DA11041, DP1 DA034989). The content is solely the responsibility of the authors and does not necessarily represent the official views of the National Institutes of Health.
